# Green gravel: a novel restoration tool to combat kelp forest decline

**DOI:** 10.1038/s41598-020-60553-x

**Published:** 2020-03-04

**Authors:** Stein Fredriksen, Karen Filbee-Dexter, Kjell Magnus Norderhaug, Henning Steen, Torjan Bodvin, Melinda A. Coleman, Frithjof Moy, Thomas Wernberg

**Affiliations:** 1 Institute of Marine Research, Nye Flødevigveien 20, 4817 His, Norway; 2 University of Oslo, Department of Biosciences, PO Box 1066 Blindern, 0316 Oslo, Norway; 3 UWA Oceans Institute & School of Biological Sciences, The University of Western Australia, Perth, Australia; 4 New South Wales Department of Primary Industries, Coffs Harbour, Australia

**Keywords:** Biooceanography, Restoration ecology

## Abstract

Kelp forests are in decline globally and large-scale intervention could be required to halt the loss of these valuable ecosystems. To date kelp forest restoration has had limited success and been expensive and unable to address the increasing scale of ecosystem deterioration. Here we developed and tested a new approach: “green gravel”. Small rocks were seeded with kelp and reared in the laboratory until 2–3 cm, before out-planting to the field. The out-planted kelp had high survival and growth over 9 months, even when dropped from the surface. This technique is cheap, simple, and does not require scuba diving or highly trained field workers. It can be up-scaled to treat large areas or even used to introduce genes from more resilient kelp populations onto vulnerable reefs. Green gravel thus overcomes some of the current major limitations of kelp restoration and provides a promising new defense against kelp forest decline.

## Introduction

**Figure 1:**
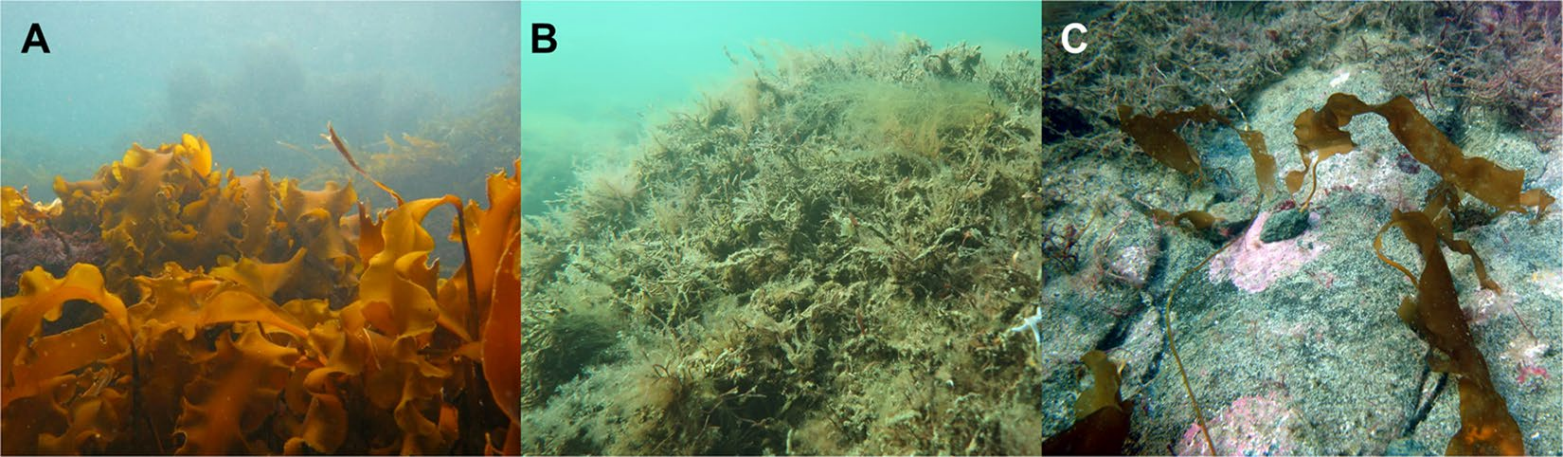
Healthy sugar kelp forests (*Saccharina latissima*, **A**) disappeared along the southern coast of Norway in 2002^47–51^. Large tracts of coast once dominated by sugar kelp now only have scattered individuals, and the seafloor is instead covered by a dense carpet of turf algae (**B**). This loss has been persistent, warranting investigation of restoration techniques – such as green gravel (**C**) – to aid recovery. Photo credits: K. Filbee-Dexter (**A**), T. Wernberg (**B**), S. Fredriksen (**C**).

Habitat deterioration and destruction are among the most pervasive threats to ecological function, and are occurring in coastal areas globally^1–3^. There is urgent need for novel solutions to combat habitat loss and promote resilience in marine ecosystems. Kelp forests are widely distributed marine habitats characterized by large habitat-forming brown seaweeds^4^ that support high primary and secondary production^5,6^, as well as a high diversity of associated plants and animals^7–9^. Direct services derived from kelp forests include shoreline protection from erosion, carbon storage, and valuable fisheries^10,11^. Kelp forests are declining globally^1,12^ and are increasingly being replaced by flattened and degraded turf reefs (Fig. 1)^11^. These transformations are linked to effects of human activities such as ocean warming and eutrophication, and have serious consequences for coastal societies that rely on resources provided by these habitats. To date, however, there has been little to no natural recovery of degraded kelp forests and projections for future losses in some areas are alarming^13^, prompting possible active interventions including restoration and rehabilitation.

**Figure 2:**
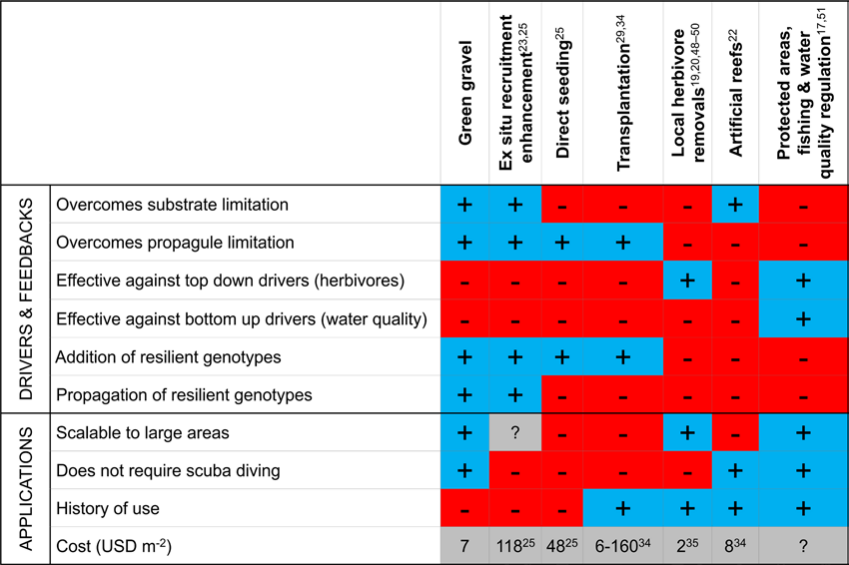
Strengths of kelp forest restoration approaches. Existing kelp restoration methods and summary of their effectiveness against various drivers of kelp loss and feedbacks preventing recovery, and highlighting strengths and weaknesses in their application^48–51^.

Current efforts to restore kelp forests are focused on high investment approaches such as top-down strategies including predator protection, or have been restricted to small scales (Fig. 2)^14,15^. Some of the earliest and most successful restoration strategies involved fishing and hunting restrictions, including the establishment of marine reserves to increase top predators and reduce populations of sea urchins, a key grazer of kelp^16,17^. Similarly, small areas of kelp forests have recovered after localized sea urchin removals by commercial harvest, ‘quick liming’ the reef, or culling by divers^18–21^. Artificial kelp forests have also been successfully created by adding boulders to sandy bottoms to offset kelp loss caused by coastal development in adjacent areas^22^. Seeding techniques and methods to enhance natural recruitment have been proposed for degraded reefs with low propagule supply^23,24^ and used to successfully establish self-maintaining *Cystoseira* forests on 25 m^2^ reefs in the Mediterranean^25^. Transplanting adult kelps has also been explored on small scales in some countries, with mixed results^21,26^. One of the most successful seaweed restoration programs has been for the canopy-forming fucoid alga *Phyllospora comosa*, which was successfully transplanted into areas of Sydney Harbour where it had gone locally extinct^27,28^. Within a single generation it formed self-sustaining populations^29^.

However, despite these few successes, marine restoration is generally challenged by the difficulties involved in working underwater, species with complex multi-phasic life histories, and large scales of loss^14^. Donor plants must be kept *in situ* in dynamic wave-exposed habitats long enough for reproduction to occur. This involves complex engineered structures to be attached to the reef, which are often vulnerable to storms and waves^29^. Moreover, deployment of such structures is labour and skill intensive and typically involves SCUBA diving under challenging conditions with strict work health and safety requirements. These challenges have limited the application and success of marine restoration, particularly for laminarian kelp forests. In addition, the dynamics of kelp loss has shifted in many regions from top down impacts such as destructive sea urchin grazing to climate-driven impacts such as direct mortality from extreme temperatures or expansion of algal competitors and competition for space^11,30,31^. This shift is rendering top-down focused restoration approaches, such as marine protected areas, largely ineffective for many systems.

Here we developed and tested “green gravel”, a novel restoration technique for kelp forests, which overcomes many limitations of current approaches. Restoring reefs using green gravel requires little investment and provides potential pathways to propagate resistant genotypes that could ‘future proof’ vulnerable kelp forests to future stress. Moreover, the technique is applicable to both laminarian and fucoid kelp forests.

## Results

**Figure 3:**
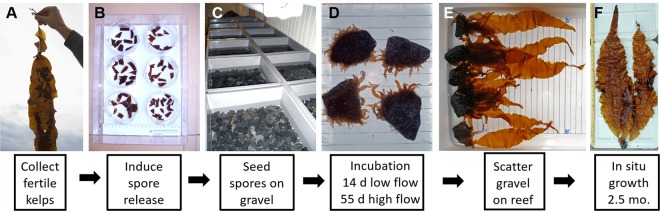
Work flow for making green gravel. Fertile plants are collected (**A**) and reproductive tissue isolated for zoospore release (**B**). Small rocks are seeded in trays by adding spore solution (**C**). After a few weeks small sporophytes are visible (**D**). Green gravel are scattered on the reef (**E**) where they continue to grow (**F**). Photo credits: H. Steen, all photos.

**Figure 4:**
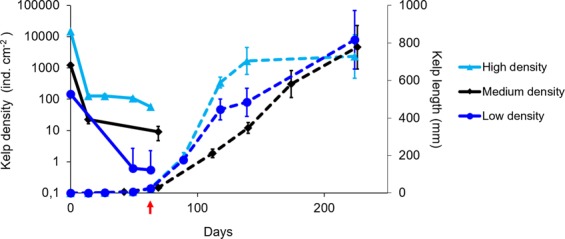
Kelp (*Saccharina latissima*) density (solid lines) and length (dashed lines) (mean ± SE) on green gravel over time during rearing in laboratory tanks and transplantation to raceway (arrow).

Fertile kelp plants (*Saccharina latissima*) were collected (n = 10–15; Fig. 3A) and sporogenic tissue was excised and put into petri dishes for zoospore release. Kelp zoospores were added to small rocks (gravel according to the Wentworth scale) (Fig. 3B,C) in different concentrations, which resulted in high, medium and low starting densities (14814, 1235 and 148 kelp individuals cm^−2^), respectively. Gametophytes fertilized following seeding and kelp sporophytes were visible at 14 days (Fig. 3D). In all three seeding densities, subsequent sporophyte abundances declined exponentially as the kelps grew. On average, the seeded kelp grew 3.4 ± 0.2 SD mm d^−1^ in the laboratory, reaching a maximum length of >90 cm after 224 days in a raceway (Fig. 4). There were no differences in growth among starting densities (kelp length: two-way ANOVA; time and density, *F*_*2,60*_ = 2.29, p = 0.11). Rates of kelp density decline were significantly different (one-way ANOVA; density, *F*_*2,29*_ = 137.2, p < 0.05), with greater rates of decline in high compared to low and medium starting densities (Tukey’s post hoc p < 0.05).

**Figure 5:**
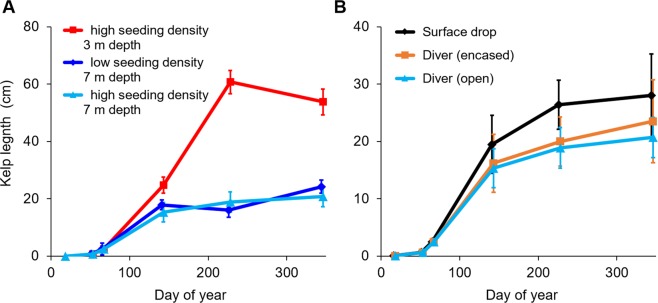
Kelp (*Saccharina latissima*) plant length (mean ± SE) on green gravel out-planted in the field, using three different deployment techniques at 7 m depth (**A**) and at different depths using high and low seeding densities (**B**).

After being reared in the laboratory and raceway for 69 days (Fig. 3E,F), the green gravel was out-planted to field sites near reefs previously dominated by kelp forests (Fig. 1). Three methods of out-planting were tested: dropping the gravel with kelps from the surface, having a diver take them down placing them either encased in trays or in open plots, directly on the seafloor. The kelps on the out-planted green gravel continued to grow in the field, reaching lengths of 20 to 35 cm after 342 days at 7 m depth (Fig. 5A,B), which was smaller than the 54 cm attained at 3 m depth (Fig. 5B, *t*-test; depth, *t*_*1,3.6*_ = 36.5, p = 0.045). Kelps on green gravel placed on the seafloor by divers (in open plots and in trays) grew at similar rates compared to kelp on green gravel dropped from the surface to the same depth (7 m), and there were no differences in kelp length in the field among deployment methods (two-way ANOVA; time and deployment, *F*_*2,80*_ = 1.63, p = 0.202) (Fig. 5A) or starting densities (two-way ANOVA; time and density, *F*_*2,90*_ < 0.01, p = 0.994) (Fig. 5B). At 85 days after out-planting, 60% of the gravel in trays and 53% of the gravel in open plots retained kelp, and between 85 and 203 days 100% of the gravel retained kelp. The plants showed a high degree of self-thinning in the field, changing from a dense cover of many small sporophytes in the laboratory, to many kelps on each piece of green gravel at the time of out-planting, to only one or two large individuals on each gravel at the end of the experiment (Fig. S1).

**Figure 6:**
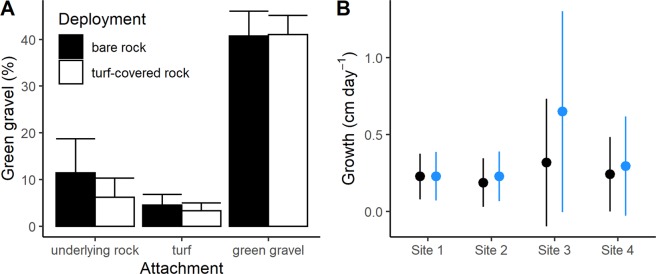
Proportion of (mean + SD) of individual gravel pieces where kelp had attached to the underlying rock, the surrounding turf algae, or remained attached only to the green gravel, 4 months after they were placed in cleared (bare rock) or untouched (turf-covered rock) plots (**A**). Growth (mean ± SD) of kelp on green gravel in cleared (bare rock) and untouched (turf-covered rock) plots across study sites (**B**).

Growth and retention of green gravel on degraded rocky reefs was tested in an additional field experiment over 4 months (early February to May). Green gravel was created following the same protocol as above and deployed at 4 sites in plots with high cover of turf algae or bare rock (Fig. 1C). Across all sites, 18.5% (±7.1 SD) of the gravel remaining after 4 months held a kelp plant that had overgrown the rock and attached to the underlying bare rock or turf (Fig. 6A). Some plants (11.8% of those showing attachment) even penetrated through dense turf to attach on the underlying rock. There was no significant difference between attachment success for kelps on green gravel deployed on turf or on bare rock (linear mixed effects model; fixed factor substrate; *F*_*1,12*_ = 0.296, p = 0.596), although we observed that gravel was retained better in turf than on bare rock. Kelps on gravel deployed onto bare rock grew slightly slower (0.22 ± 0.02 cm d^−1^) compared to gravel deployed onto turf-covered rock (0.30 ± 0.05 cm d^−1^) (linear mixed effects model; fixed factor substrate; *F*_*1, 119*_ = 8.80, p = 0.004) Fig. 6B)). Final densities of kelp on green gravel averaged 17 ± 8 m^−2^ in turf plots and 16 ± 9 m^−2^ in bare rock plots, compared to initial densities of 24 m^−2^ in both types of plots. This shows that seeded juvenile kelps can eventually overgrow the green gravel and attach to the surrounding substrate or underlying rock, which suggests the plants could be retained on these reefs.

Based on our time and personnel requirements during these field trials, we estimate that 116 kg green gravel (the amount created but not used in this study) will be enough to restore 314 m^2^ reef [5 granite gravel m^−2^ @ ~75 g gravel^−1^]. Producing this quantity of green gravel required 1.5 m^2^ wet laboratory tanks, 40 hours of laboratory time for culturing and maintaining tanks, and would require approximately 14 hours of field time for collection and deployment. Equipment, fuel and materials, not including facilities, vessel rentals, and bench fees, were approximately 500 USD. Using hourly rates of 30 USD h^−1^, which are comparable to other studies, this totals 6.75 USD per m^−2^ of restored reef.

## Discussion

Kelp forest restoration is currently challenged by the changing nature and increasing scale of habitat transformation of temperate reefs^1^. We suggest that green gravel could be an alternative to traditional restoration techniques because it addresses many of the impacts on kelp forests that top-down approaches (e.g., marine protected areas) are largely ineffective against. In this context, green gravel overcomes issues of both propagule limitation and lack of hard substrate, two key feedbacks that are preventing recovery following recent kelp forest collapses^32,33^. Changes in ocean conditions associated with these collapses (e.g., warming, altered currents, marine heatwaves) are projected to intensify in the future, creating a strong imperative to transform restoration strategies to be effective against both historical drivers of loss and these new dynamics.

Green gravel shows promise as an efficient and cost-effective approach with great potential for upscaling to tackle the increasing scale of kelp loss. The cost of this tool (7 US$ m^−2^) is smaller or comparable to other restoration techniques including methods of transplantation and recruitment enhancement (Fig. 2). However, unlike manipulating adult kelps or larger artificial substrates, green gravel is easily handled and transported in manageable batches and large volumes can be deployed across large areas without installation, overcoming important logistical obstacles to marine restoration. Moreover, we showed that handling did not prevent the eventual successful growth of kelp plants on the gravel (despite natural self-thinning), and there was similar growth among seeding densities, suggesting the method is robust to partial kelp mortality from rough treatment during deployment. While many kelp restoration approaches are challenged by limitations imposed by working in underwater environments, and as a consequence can only tackle small scales (Fig. 2)^18,20,21,29^, kelp on green gravel that was dropped from the surface grew at the same rate as gravel placed on the sea floor by divers. Collections of reproductive material from source populations can also be made from a vessel using rakes or other tools, where these collection methods do not compromise donor populations. This shows the upscaling potential of this technique using untrained personnel working from the surface. It also removes the requirement that personnel work underwater as this is very labour-intensive, requires substantial training investments and is governed by strict health and safety considerations. We contend that the true costs of up-scaling has been underestimated for many diving-intensive restoration approaches where diving typically is budgeted at 15–45 USD h^−1^ ^25,34^, based on volunteer or scientific divers, where commercial divers charging rates from 111 to 238 USD h^−1^ ^35^ will be required for full-scale operations in most countries.

Green gravel also circumvents the need to destructively harvest and transplant large quantities of whole adult donor plants, thereby potentially depleting and compromising other natural, potentially vulnerable, populations. Although reproductive tissue from adult plants must be harvested for zoospore release, for many species this can be achieved through partial (non-destructive) removal of sporophylls or sori (or receptacles in the case of fucoids) without damaging the primary meristem of adult plants. A priori information on genetic diversity and structure of local or surrounding adult populations is required to ensure appropriate, natural levels of genetic diversity are replicated when sourcing adult plants for zoospore release and that collections are targeted to appropriate, local donor populations.

The efficacy of green gravel remains to be tested under a broad range of environmental conditions including high grazing pressure or high wave action. For example, gravel may be quickly dispersed away from the targeted areas by high water motion. However, even in high energy environments, gravel is likely to be retained in cracks and crevices and still provide a propagule source to reseed areas where this is a limiting factor. We showed that over many months green gravel deployed in open plots remained in place and grew at similar rates and experienced similar survival to gravel in trays, and started to attach to the underlying reef, even if covered with turf algae. This is further evidence that green gravel tend to remain where delivered. Additional factors that also require more investigation include the density of gravel (and therefore adult canopy cover in restored areas) required to allow kelp forests to reach thresholds of canopy cover to overcome feedbacks maintaining turf.

A major hurdle for marine restoration and conservation^36^ is that changing ocean conditions are overwhelming the intrinsic capacity of organisms - including kelps - to adapt and survive in parts of their range^1,37,38^. This has prompted discussion surrounding novel interventions to reduce and reverse loss^39,40^. Perhaps the most promising application of green gravel lies in exploring the potential to enhance the resilience of existing but vulnerable kelp forests through assisted adaptation^40–42^. Green gravel could be seeded with selected or engineered genotypes that are resilient to stressors of interest, and easily and cost-effectively deployed into extant kelp forests across large scales and inaccessible areas. Although similar techniques involving propagating resilient individuals have been employed in terrestrial systems and aquaculture for centuries (selective breeding), the advent of sequencing technologies and the ability to identify genotypes and loci that are selected for under certain environmental conditions^43^ now makes this a reality for kelp forests. Indeed, novel genetic technologies combined with green gravel may open the possibility to design bespoke assisted adaptation strategies to boost resilience of extant kelp forests to a variety of stressors. Despite its promise, caution must be taken to avoid outbreeding depression or disrupting adaptation to non-target stressors^41^ and ecological surprises^44^, and legal and ethical implications must be considered^39^. A large body of research is required before such conservation strategies could be sensibly developed with green gravel and used in natural settings^40^.

As a restoration technique, green gravel may work particularly well in combination with other approaches such as establishing protected areas, or reducing stressors (e.g. harvesting regulations or water quality improvement). This would address multiple different drivers of kelp loss (e.g., pollution, run-off, coastal development, and top down impacts). As such, green gravel should be added to the suite of tools available to combat declining kelp forests. More broadly, the green gravel concept has the potential to be a globally applicable marine restoration method that transcends species and systems.

## Materials and Methods

Sugar kelp (*Saccharina latissima*) were seeded directly onto small (~3 × 3 × 3 cm) granite rocks, then grown in the laboratory and transplanted into the field (see Fig. 3 for work flow). Between 10 and 15 fertile individuals of *S. latissima* with visible sori were collected in autumn (November) from reefs outside Flødevigen Research Station, in southern Norway (58.423° N, 8.757° E). Zoospores were released using standard protocols for laminarian kelps^45^. Specifically, the laminae were rinsed in fresh water to clean them of epiphytes. The sori were cut out and left in a moist paper towel in a fridge for 24 hours, then cut into smaller pieces and put in sea water to induce spore release. The resulting spore solution was separated into high concentration (15000 spores ml^−1^) and low concentration (150 spores ml^−1^) solutions, by counting the number of spores under a microscope. Three separate laboratory trials were conducted, each with a distinct starting kelp density (14814, 1235 and 148 kelp individuals cm^−2^, created using high, high and low concentration solutions respectively). For all trials, spore solutions were added to boxes (45 × 45 cm) containing gravel (2–5 cm in diameter) and submerged in flowing seawater that was pumped from 75 m depth. For the first 2 weeks the water flow was approximately 20 L per hour. After 2 weeks the germlings were visible and the flow was increased to 60 L per hour. Constant light (25 µmol m^−2^s^−1^) and temperature (11 °C) conditions were maintained during the incubation period. The kelp density and length of the longest plant on each of five randomly chosen pieces of gravel from each box was measured 4 times over the incubation period. After 2.5 months green gravel were transplanted into the sea outside Flødevigen Research Station (Fig. 3). The average maximum plant length at the time of out-planting was approximately 2.5 cm. A subset of the green gravel were transplanted into a raceway (3.6 m × 0.41 m × 0.17 m flume with flow-through seawater; water level = 0.135 m; with 60 L per hour flow and same light and temperature as above) in the laboratory and measured over 224 days.

To test the effectiveness of different transplantation techniques the gravel was out-planted using three different methods: (1) divers placed the gravel in open boxes (30 × 40 cm) on the seafloor at 7 m depth, (2) divers laid the gravel in open plots on bare rock at 7 m depth, and (3) gravel were dropped to 3 m and 7 m depth from a boat. Gravel was dropped from the surface by overturning a tray of gravel over the side of the boat, while a diver marked the landing site. Out-planting was done in early winter (January) and growth measured in April, June and October, by collecting gravel from each treatment and measuring the maximum plant length on each gravel (*n* = 10). The field site was located in a semi-protected area with patchy kelp (2–20% canopy cover) and turf-dominated reefs and mainly coarse sedimentary substrate. For additional detail see^46^.

To test whether gravel could be retained on rocky reefs, divers transplanted green gravel onto bare and turf-covered reefs at 6 m depth at 4 sites in early winter (February 2019). At each site a handful of gravel was placed on the seafloor inside 3 cleared 0.25 m^2^ plots or inside 3 ‘open plots’ (see above) with high turf-algae cover (n = 5–7 gravel in each plot). Gravel was covered with small kelp sporelings <2 cm length. Cleared plot treatments were created by removing all turf algae with a scraper from a 0.25 m^2^ quadrat, leaving only bare rock and encrusting coralline algae. We revisited sites in May, measured maximum plant length on each gravel and recorded if any kelps had attached to the underlying rock or turf algae.

The cost effectiveness of the green gravel method was assessed by tallying up the person-hours and materials used to produce and culture 116 kg green gravel, corresponding to a full raceway, as well as the estimated person hours, fuel and materials required for field deployment of this quantity when dropped from the surface. Bench fees, vessel, vehicles and other fixed infrastructure were not included and neither was subsequent monitoring. This approach is consistent with similar cost assessments for other seaweed restoration techniques (Fig. 2).

### Statistical analyses

Data were analysed by ANOVA, Linear Mixed Models, and t-tests performed in R (package lme). In the second field experiment, substrate (turf and bare rock) was treated as a fixed factor and study sites and replicated plots were treated as random factors with plots nested within sites. Assumptions of normality and homoschedasticity were assessed by visual inspection of residuals and error distributions.

## Supplementary information

Supplementary Information.

## Data Availability

Data are available from the Institute of Marine Research data repository.
