# Supplementary material for: Green gravel: a novel restoration tool to combat kelp forest decline
Source: Sci Rep. 2020 Mar 4;10:3983. doi: 10.1038/s41598-020-60553-x (PMC7055217; doi:10.1038/s41598-020-60553-x)
Supplement: Supplementary file 1 — Supplementary Information. [file 41598_2020_60553_MOESM1_ESM.pdf]

Supplementary material

Green gravel: a novel restoration tool to combat kelp forests decline

Stein Fredriksen,[1,2*] Karen Filbee-Dexter,[1,3] Kjell Magnus Norderhaug,[1] Henning Steen,[1] Torjan Bodvin,[1]† Melinda A. Coleman,[4] Frithjof Moy,[1] Thomas Wernberg[3*]

[1] Institute of Marine Research, Nye Flødevigveien 20, 4817 His, Norway
[2] University of Oslo, Department of Biosciences, PO Box 1066 Blindern, 0316 Oslo, Norway.
[3]UWA Oceans Institute & School of Biological Sciences, The University of Western Australia, Perth, Australia
[4]New South Wales Department of Primary Industries, Coffs Harbour, Australia

*Corresponding authors: stein.fredriksen@ibv.uio.no, thomas.wernberg@uwa.edu.au
†Deceased

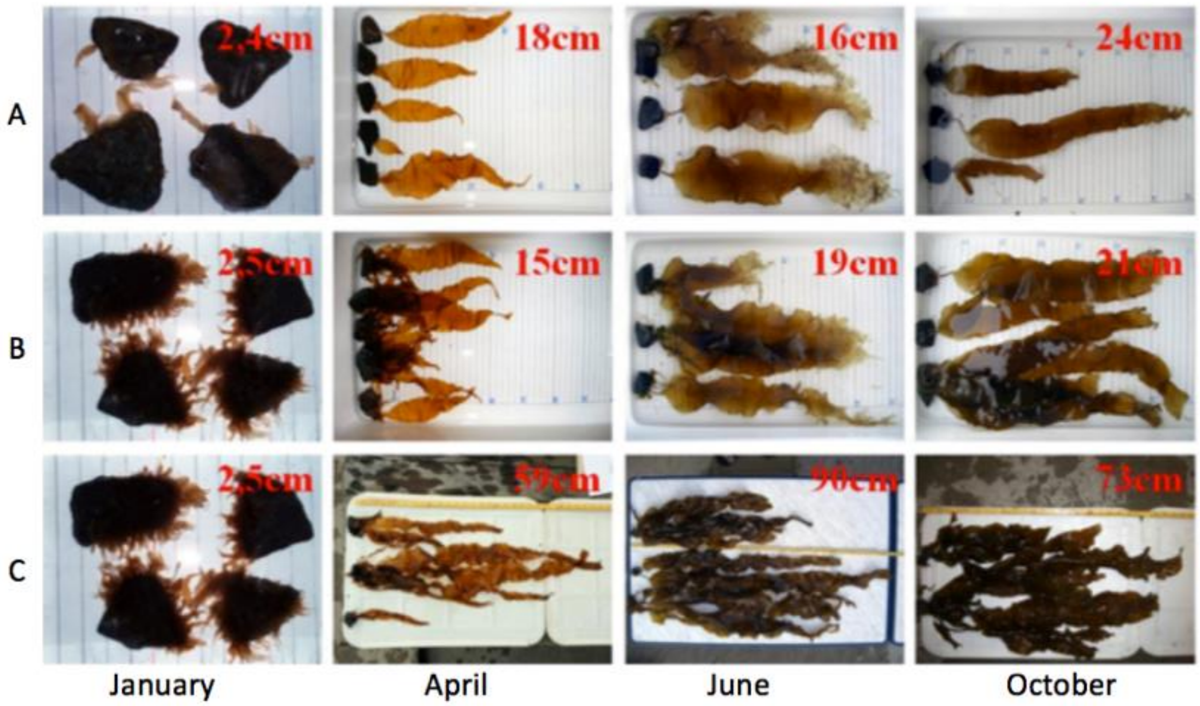

Figure S1. Green gravel seeded with kelp (*Saccharina latissima*) and held in the lab and kelp plants after growth in the field in April, June and October at 7 m depth. Row A: Gravel with low seedling density, B: Gravel with high seedling density. C: Gravel from in-door seedling raceways. Average maximum plant length from 5 gravels is indicated in red on each photo.
